# Knowledge and Acceptability of Fecal Microbiota Transplantation Among Patients, Caregivers, and Health Care Providers in Ethiopia

**DOI:** 10.1093/ofid/ofaf676

**Published:** 2025-11-04

**Authors:** Brandie Banner Shackelford, Kiya Kedir, Ahmed Babiker, Bizunesh Sintayehu, Abel Abera Negash, Alemseged Abdissa, Workeabeba Abebe Taye, Eyob Beyene, Michael H Woodworth, Monique M Hennink

**Affiliations:** Gangarosa Department of Environmental Health, Rollins School of Public Health, Emory University, Atlanta, Georgia, USA; Armauer Hansen Research Institute, Addis Ababa, Ethiopia; Division of Infectious Diseases, Department of Internal Medicine, Rush University Medical Center, Chicago, Illinois, USA; Armauer Hansen Research Institute, Addis Ababa, Ethiopia; Armauer Hansen Research Institute, Addis Ababa, Ethiopia; Armauer Hansen Research Institute, Addis Ababa, Ethiopia; Department of Pediatrics and Child Health, Addis Ababa University, Addis Ababa, Ethiopia; Department of Internal Medicine, Addis Ababa University, Addis Ababa, Ethiopia; Division of Infectious Diseases, Department of Medicine, Emory University School of Medicine, Atlanta, Georgia, USA; Hubert Department of Global Health, Rollins School of Public Health, Emory University, Atlanta, Georgia, USA

**Keywords:** antibiotic resistance, fecal microbiota transplantation, malnutrition, microbiome, multidrug-resistant organism

## Abstract

**Background:**

Malnutrition and antimicrobial-resistant infections are major causes of morbidity and mortality in low-income countries. These conditions have been associated with the gut microbiome, although little is known about the acceptability of microbiota therapies such as fecal microbiota transplantation (FMT). We explored the acceptability of FMT among health care providers (HCPs) and patients in Addis Ababa, Ethiopia.

**Methods:**

In this qualitative study, we purposively sampled patients with bacterial infections and acute malnutrition, caregivers, and HCPs at two hospitals. Eight focus group discussions were held. Amharic and English discussion guides covered knowledge of FMT and perceived barriers or facilitators for uptake. Data were transcribed and translated into English when necessary. MAXQDA software was used for a thematic analysis, with trained researchers closely reading transcripts to identify issues, develop a codebook, iteratively code data, and assess intercoder agreement. Description, comparison, and categorization were conducted to discern core themes, and validity checks ensured that findings were grounded in the data.

**Results:**

HCPs indicated a general willingness to prescribe FMT, provided that there was sufficient evidence supporting its efficacy and safety and they were confident on patient adherence. Patient acceptability of FMT was categorized along a continuum from those who were unconvinced, persuadable, amenable, and accepting of salvage treatment.

**Conclusions:**

FMT may be acceptable for HCPs and patients in Addis Ababa, although interventions are needed to enhance acceptance among some groups, such as marketing it as standard medication, obtaining endorsement by religious leaders, providing multiple formulations, and/or providing thoughtful health communication.

## Teaser

This qualitative study explored knowledge and acceptability of fecal microbiota transplantation (FMT) amongst patients/caregivers and health care providers in Addis Ababa, Ethiopia. While most providers supported FMT given evidence of safety and efficacy, patient acceptance varied along a continuum from unconvinced, persuadable, amenable, to acceptance as salvage treatment – highlighting the need for tailored messaging, religious engagement, and flexible formulations.

## Graphical Abstract

**Graphical Abstract:**
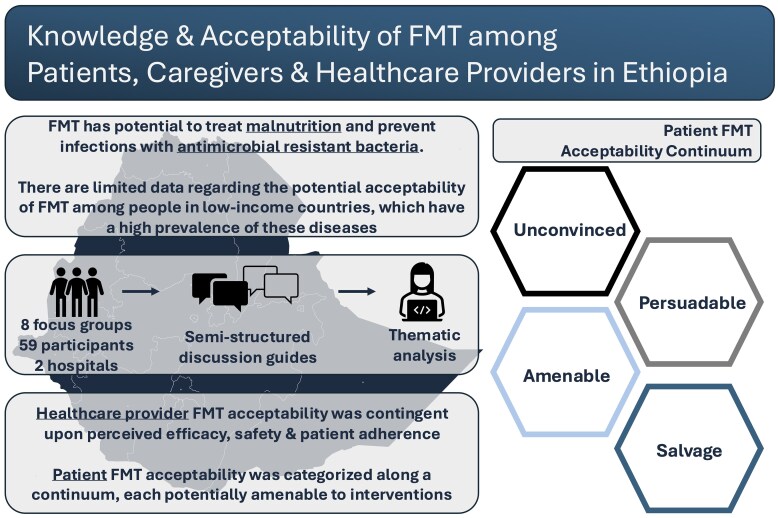

Malnutrition and antimicrobial-resistant (AMR) bacterial infections are major causes of excess morbidity and mortality in low-income countries (LICs) and are associated with intestinal microbiome dysbiosis [1–5]. The intestinal tract is a reservoir for enteric AMR bacterial pathogens, with LICs bearing a high burden of multidrug-resistant organism colonization [6]. Several intestinal bacterial taxa have been associated with reduced risk for multidrug-resistant organism colonization and malnutrition [1, 2, 7]. These findings have bolstered enthusiasm for the study of microbiota therapeutics (MT) such as fecal microbiota transplantation (FMT) as an intervention for AMR infection and malnutrition [6, 7].

FMT involves the transfer of complex fecal microbial communities derived from healthy screened donors [8]. FMT can be delivered to the upper gastrointestinal tract via capsules or a nasogastric tube or to the lower gastrointestinal tract via retention enema, colonoscopy, or sigmoidoscopy [9]. Methods to isolate and culture these microbial communities may facilitate scalable manufacture of preparations that are easier to produce and administer [10]. Despite reported efficacy of FMT for eradication of AMR bacteria colonization and malnutrition unresponsive to food-based solutions, little is understood about the microbiome composition in Ethiopia [11–13]. We are unaware of prior work evaluating the acceptability or barriers to use of MT in LICs more broadly. This knowledge gap impairs the translational development of and access to MT for people living in LICs.

Our study builds on existing evidence from middle- and high-income countries by exploring patient and health care provider (HCP) emic perspectives on FMT in an LIC. We aimed to explore the knowledge and acceptability of FMT in patients and HCPs in Addis Ababa.

## METHODS

The study was conducted in two hospitals in Addis Ababa, Ethiopia. Tikur Anbessa Specialized Hospital, colloquially known as Black Lion, is the largest government referral hospital in the country. It has 600 beds and serves a diverse patient population from all regions in Ethiopia [14]. As a specialty care facility, it includes senior physicians and infectious disease fellows. The All-Africa Leprosy, Tuberculosis, and Rehabilitation Training (ALERT) Comprehensive Specialized Hospital has 377 beds and primarily serves patients from Addis Ababa. There is a high prevalence of antibiotic use at ALERT [15], making it suitable for exploring FMT treatments. Together, these hospitals offer access to a highly varied patient population and HCPs with expertise in infectious disease, making them suitable sites for this study.

Study participants included patients or their primary caregivers and HCPs from study hospitals. Eligible patients were adults with bacterial infection being treated with systemic antibiotics (enteral or parenteral) and parents/caregivers of children aged <18 years with acute malnutrition—conditions potentially treatable with MT. Potential participants were screened for eligibility via hospital registration forms and physician referrals. HCPs who routinely treat such conditions were recruited through peer referrals, announcements at division meetings, and hospital notices. Research staff purposively selected diverse patients/caregivers and HCPs based on demographics and professional roles to ensure a broad range of perspectives (Supplementary Material 1). For all participants, oral consent was obtained during recruitment, followed by a written Amharic consent form. Exclusion criteria included the inability to attend a focus group discussion (FGD), refusal of informed consent, or unwillingness to be recorded. Research staff confirmed eligibility, invited participants, and informed them of the time and location of the FGDs at the hospital.

Data were collected from April 2024 to April 2025 through eight FGDs comprising six to eight participants across both hospitals, with a total of 59 participants. Five patient/caregiver FGDs were conducted: three with men and two with women. Three FGDs were conducted with HCPs with various roles (eg, physicians, pharmacists, nurses, fellows). These HCP FGDs were mixed gender since discussions centered on views of a hypothetical, unavailable therapeutic. Saturation was achieved in patient and HCP populations within three FGDs, whereby no more new issues were raised. This aligns with empirical evidence whereby saturation can be reached after three to six FGDs [16]. The consistency of issues raised across focus groups—despite diverse demographics and professions, as well as the limited knowledge of FMT and the low health literacy of patients—contributed to reaching saturation with few focus groups. Thus, the data collected were adequate for the study.

FGDs explored perceptions of FMT, capturing shared and divergent views. Conducted in private hospital spaces, the sessions were led by experienced qualitative researchers. Refreshments were offered to participants as an incentive. Discussions were held in Amharic (patients/caregivers) or English (HCPs) and audio recorded with participants' consent, and contributions were anonymized via numbers as pseudonyms. Semistructured discussion guides (Supplementary Materials 2 and 3) were translated into Amharic. Participants were first asked open-ended questions on current treatments for infections/malnutrition. Next, the concept of MT was introduced, including FMT and live-cultured bacterial therapeutics. Participants were asked about prior knowledge and acceptability, formulation preferences, and influences on adoption. Although the term MT was used for accessibility, questions explicitly distinguished between FMT and other MTs to assess any differential acceptability.

FGD recordings were translated into English where necessary, transcribed verbatim, and deidentified before analysis in MAXQDA (Verbi GmbH). Translations were reviewed for accuracy by two researchers. A thematic analysis was conducted involving iterative tasks: reading transcripts to identify issues, developing a codebook with inductive and deductive codes, assessing intercoder agreement, and coding data in MAXQDA. Coded transcripts were analyzed using description to define key issues; comparison to identify any patterns by gender, patient/HCP, or hospital; and categorization to identify broader groups reflecting similar perceptions of MT/FMT. Consistency checks, including negative/deviant case analyses and cultural validation by key informants [17], ensured that categories were fully supported by data. The study was deemed exempt from ethical approval by the Emory University Institutional Review Board (00009196) and Ethiopia (PO66/22).

## RESULTS

The results describe the variation in participants' knowledge of MT, their acceptance of MT and FMT, and their preferences regarding FMT formulation. Comparisons across gender revealed no consistent patterns.

### Knowledge and Perceptions of MT and FMT

Some HCPs had never heard of MT before the FGD, while others were familiar with it through scientific literature, academic lectures, social media, or a medical exchange program. Those previously aware knew that MT could be made from stool, and they reported that it could be used to treat conditions such as infections, inflammatory diseases, and autism. HCPs felt that MTs that were not stool derived would be easier to understand, explain to patients, and therefore accept than FMT. This was attributed to familiarity with the use of beneficial bacteria in vaccines or yogurt. Some HCPs emphasized the ethical importance of disclosure (ie, that FMT is stool derived) to maintain patient trust, while others felt that complete transparency was not necessary and suggested listing specific microorganisms instead. For example:

It is not ethical to prescribe medication without telling [patients] from what it is prepared. . . . New ideas are always a challenge. Until the community gets used to the idea of such medicine and awareness level increases, I don’t think telling the community every detail is necessary. . . . Once they start to see the benefit of the medicine, slowly introducing what it is made of may work. From the start, telling them that it is made from stool will not be accepted. . . . Using a taboo word at the beginning and spilling all the details will be an obstacle.

Patients/caregivers reported either no knowledge of MT or some exposure to it from the media. They expressed little preference between non-stool-derived MT and FMT. While some believed that they had a right to be informed about the composition of their treatments, others felt that disclosure was unnecessary or unwanted. One male participant explained, “We don’t know anything about the medicines we have been taking. . . Nobody investigates or cares to know the content. What we care about is—does it treat our ailment?” Another said, “Since it is a treatment, we shall take as it is. Knowing might affect us.” Overall, participants agreed that the acceptability of MT and FMT is likely to increase over time as these treatments become more familiar and people become aware of successful outcomes. For example, “In our culture people ask and learn gradually thus they might be willing after learning from others.”

### HCP Acceptability of FMT

HCP acceptability to prescribe FMT fell into dichotomous groups: those unwilling to prescribe FMT and those willing to prescribe it, contingent upon demonstrated efficacy, safety, and patient adherence. HCPs unwilling to prescribe FMT expressed feelings of disgust toward stool-based treatments and stated that they would not prescribe a treatment that they would not take themselves.

HCPs who were willing to prescribe FMT were eager to learn about FMT as an alternative treatment to antibiotics. For example, “[The] antibiotic resistance we are seeing is really concerning. We always wish we had options other than antibiotics.” This group wanted more information about its efficacy and safety as compared with antibiotics, particularly in reducing infection reoccurrence and AMR. Some wanted evidence that FMT was more efficacious than antibiotics, whereas others were willing to prescribe if it was equally effective. An HCP noted, “If it is even equally successful with other antibiotics. 100% I would prefer over antibiotics. It is highly likely that I will use if there is concrete information like side effect and efficacy.” HCPs were concerned that transplanted donor stool could cause other illnesses, especially in patients with comorbidities, such as those who are immunocompromised. Some HCPs were also concerned about donor age, while others emphasized that donor-patient similarity was important, particularly in terms of diet. Several HCPs from ALERT emphasized the importance of research conducted in comparable settings, with one noting, “If there is research done its mostly on White people. When it comes to our country, race and demographic might be an issue and make us hesitate to prescribe.” HCPs felt that efficacy and safety should be demonstrated through endorsement by respected regulatory authorities, such as the World Health Organization (WHO) and the Food and Drug Administration (FDA).

HCPs indicated that perceived patient acceptance and subsequent adherence would influence their decision to prescribe FMT. Some believed that only patients who were chronically ill would be willing to undergo the treatment, while others thought that certain patients would prefer death over FMT. There was also concern that patients would ultimately reject FMT in favor of more conventional treatments. Furthermore, HCPs emphasized that patient adherence is dependent on the availability and affordability of FMT. For example:

When a drug is made there are factors that limit its use. For example, cost is one factor. I will not prescribe a drug that is not affordable. It is no use if one does not buy and use it . . . availability should also be considered. Also, community acceptance is an issue. If the community does not accept it there is no use prescribing medicine, they don’t buy. So, we eventually shift to ones they prefer to use. Just like we are convinced, the community must also be convinced. So, if it has community acceptance, it is affordable and available, if it has followed scientific procedures we are likely to prescribe.

### Patient Acceptability of FMT

**Figure 1.**
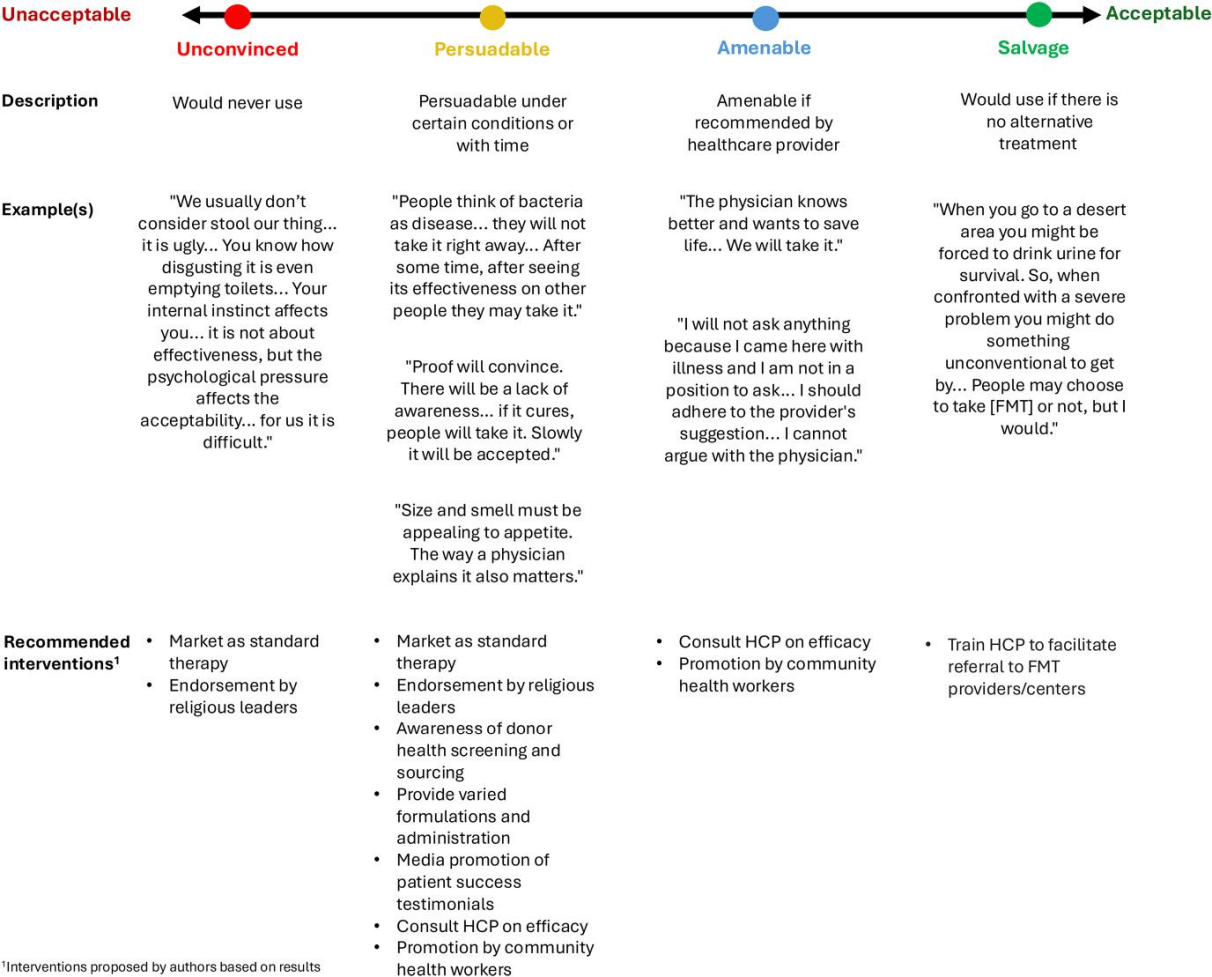
Continuum of acceptability for fecal microbiota transplantation (FMT) among patients and caregivers in Addis Ababa.

Patient/caregiver acceptability of FMT was categorized into four groups along a continuum from being unconvinced by FMT, persuadable to use it, amenable to HCP recommendations, and as salvage treatment, as shown in Figure 1 and described in turn.

#### Unconvinced

At one end of the continuum of acceptability were patients/caregivers who were unconvinced by FMT, indicating that they would never undergo the treatment. They expressed feelings of disgust knowing that FMT was made of stool. Others indicated religious influences on using FMT, with some objecting to the use of fecal material in medicine, stating that they could not use FMT if it were prohibited by their religion. An HCP elaborated on these religious concerns, stating that this could be particularly relevant to Muslim patients, since “any fluid coming out of the body defiles a person and requires purification.”

#### Persuadable

The next group along the continuum were patients/caregivers who expressed reservations regarding FMT but indicated that they could be persuaded to use it if their concerns and preferences were adequately addressed. A primary concern for this group was the efficacy of FMT. Participants said that they could be convinced to use FMT by scientific research on its efficacy, by information from their HCP, and/or by hearing the positive experiences of community members who had used FMT. One stated that she would be persuaded if she saw others being cured, even if it conflicted with her religious beliefs: “I am Muslim . . . let us say I am ill, and an Orthodox friend may come and tell me I will be cured if I go to Tebel. I may see others cured. I want to be cured so I will take it.”

These patients reported that they could be persuaded with more information on the potential adverse effects of FMT, as well as safety measures involved in its production, including donor screening. Some patients expressed interest in knowing donor characteristics such as diet, nationality, age, and/or weight, while others felt that this was unnecessary if the donor was healthy. Others were persuadable if FMT was “short act[ing]” and/or a “permanent solution” as compared with conventional treatments. Persuadable patients often had preferences on the formulation of FMT, but there was no commonly preferred route of administration (see FMT Formulation Preferences section). Other patients/caregivers reported that they could accept FMT depending on its cost and availability in local pharmacies. Patients stated that FMT should “be available in every pharmacy. In most of our government pharmacies important drugs are out of stock.”

#### Amenable

The third group of patients/caregivers was amenable to use FMT if HCPs recommended it. Some stated that they trusted their HCPs' clinical judgment: “I wouldn’t ask any question. If I had better knowledge, I could treat my child at home. I came here because I know physicians have more knowledge, so I do what he suggested. . . I trust him because for sure there is some research done.” Others, however, highlighted a power dynamic between HCPs and patients, making them reluctant to question medical guidance: “When we ask questions, some physicians get angry. . . We just say amen to anything the doctor says.”

#### Salvage

A fourth group would accept FMT as salvage therapy after exhausting other treatments, particularly in cases of chronic or critical illness with “no other option.” One patient explained, “It may be accepted when a person is very sick and desperate to get well. Some are so desperate they say I will juice up the poop and drink if it helps.” Parents with children who are critically ill expressed desperation and demonstrated a proactive approach toward FMT, emphasizing their willingness to do “anything it takes” to support their children's recovery.

### FMT Formulation Preferences

HCPs and patients exhibited diverse preferences regarding FMT formulation, although individual preferences were facilitators of acceptability only among persuadable patients. Participants preferred shorter and less frequent treatments due to concerns about long-term adherence, as patients reported forgetting to take medications consistently. To overcome adherence issues, several patients/caregivers recommended that treatment be administered in a health care facility to increase compliance.

Some participants preferred oral administration. Among HCPs, this preference was driven by concerns about stressed hospital resources (eg, staff, time) and sterilization challenges with other methods. Among patients/caregivers who preferred oral administration, some preferred capsules because they mask taste. Others voiced substantial distrust in capsules, worried about not seeing the contents and the potential health risks of the “plastic” covering. The aesthetic qualities of pills were also important to patients/caregivers, with preferences for small and visually appealing pills, and color preferences varied. Other participants preferred nonoral routes of administration, such as suppositories. These were preferred to avoid feelings of disgust related taste and odor. For child patients, participants proposed administering FMT to children via syrup with various flavors to accommodate a range of preferences.

## DISCUSSION

Most previous studies on patient [18–29] and HCP [19, 30–40] knowledge and perceptions of FMT relied on quantitative surveys, with relatively few [25, 41–43] employing qualitative methodologies that explore the full breadth of acceptability. Our results identified that patient/caregiver acceptability of FMT fell along a continuum of acceptability in Ethiopia, with each group requiring tailored interventions to facilitate FMT adoption (Figure 1).

Those patients/caregivers who were unconvinced about FMT due to concerns about disgust may overcome their hesitation if FMT is presented as a standard medication, with minimal emphasis on specific details. For those unconvinced because of religious objections, endorsement by religious authorities may reduce this barrier.

Patients/caregivers who reported that they could be persuaded to undergo FMT shared a range of influences that could persuade them to adopt. In addition to the interventions for unconvinced patients/caregivers, explaining protocols for donor screening [23] and providing various formulation options may increase acceptability for this group of patients [22]. Furthermore, participants indicated that they may be persuaded to undergo FMT if they knew that others in their community had been successfully cured by FMT over time. Previous studies support this, reporting that anecdotal evidence [21] and increased familiarity [19] improve FMT acceptability. Therefore, patient testimonials in the media and thoughtful health communication by HCPs and community health workers could encourage future FMT adoption for this group.

Patients/caregivers who were amenable to FMT reported that HCP recommendation is the primary determinant of FMT adoption. This aligns with findings from previous studies of US patients with *Clostridium difficile* infection, which found that HCP endorsement was the most significant factor influencing patient acceptability [26, 31]. Potential interventions for those who are amenable could include individual consultation by an HCP as well as promotion by health extension workers at the community level.

HCP awareness and access to treatment may be the most important factor for patients/caregivers who viewed FMT as salvage treatment, as they were willing to adopt FMT. Therefore, efforts would be needed to increase HCP awareness through training programs to facilitate referral to care where FMT is provided. Patients in the United States [23], China [29], and Canada [28, 41] similarly viewed FMT as a viable option once alternative treatments were exhausted.

Perceived efficacy, safety, and adherence emerged as determinants of FMT acceptability for some HCPs, while others found only non-stool-derived MTs acceptable. Therefore, strengthening evidence of efficacy and safety [44 ] within a comparable clinical setting could increase acceptability—ideally through a randomized controlled trial investigating FMT for eradication of AMR infections [45–47] and/or treatment of malnutrition in Ethiopia. HCPs also reported that endorsement from regulatory medical authorities would increase FMT's acceptability; in the absence of formal guidance from WHO and the Ethiopian FDA, informing providers that FMT has been approved by the US FDA for treatment of *C. difficile* [48] could help build confidence to prescribe FMT.

We observed a paradox: HCPs were hesitant to recommend FMT without confidence that patients would adhere to the treatment, yet their endorsement facilitates patient acceptance. Disseminating the findings of this study to HCPs, which demonstrate that certain segments of the patient population are willing or eager to undergo FMT with HCP endorsement, may reduce this barrier. Additionally, some HCPs expressed a preference for donors who are similar to their patients in aspects such as diet. This aligns with findings from another study [32], which reported that Jordanian HCPs were concerned about the religious background of donors and dietary intake (ie, halal). Therefore, HCPs may prefer donors sourced from the same community as the patient.

The results of this study are limited to patients/caregivers and HCPs in Addis Ababa and the cultural context of Ethiopia and may not be applicable to other contexts. The perspectives of the most rural and/or economically disadvantaged populations in Ethiopia may not be represented in this study (ie, those who cannot afford transportation to a hospital in Addis Ababa for treatment). Ethiopia is ethnically and religiously diverse, and we did not directly ask questions about how specific subcultures or religions within Ethiopia view FMT acceptability; additional research is warranted in this area. Furthermore, the voices of children regarding FMT acceptability were represented only through their caregivers, since we did not include participants aged <18 years.

The study examined the knowledge and acceptability of FMT among patients, caregivers, and HCPs in Ethiopia, and it is the only one that we are aware of that has investigated this topic in any LIC. The qualitative approach employed in this study allowed us to explore the acceptability of FMT in greater detail and uncover more nuance to acceptability than other quantitative methods. A strength of our study is the purposive sampling of participants by gender, roles, and two unique hospitals. As these hospitals are primary health care facilities, they serve patients/caregivers from a range of districts across Ethiopia, capturing a diverse dataset and variability in perspectives.

## Conclusion

This study fills a gap in the literature on acceptability of FMT among patients, caregivers, and HCPs in an LIC, where AMR infections and malnutrition are urgent threats to public health. Although originally unfamiliar to patients/caregivers in Addis Ababa, participants indicated that FMT acceptance would increase with familiarity over time. Patient acceptability of FMT was categorized along a continuum from those who were unconvinced, persuadable, amenable, and accepting of salvage therapy. This continuum of acceptance suggests that a range of interventions is needed to increase FMT acceptance in Ethiopia, such as marketing FMT as standard therapy, obtaining endorsement by religious leaders, providing multiple formulations, and tailoring health communication. Non-stool-derived MTs were viewed as broadly acceptable to HCPs, while FMT acceptability was contingent upon perception of its efficacy, safety, and patient adherence.

## Supplementary Material

ofaf676_Supplementary_Data

## Supplementary Data

Supplementary materials are available at *Open Forum Infectious Diseases* online. Consisting of data provided by the authors to benefit the reader, the posted materials are not copyedited and are the sole responsibility of the authors, so questions or comments should be addressed to the corresponding author.

## References

[1] Smith MI, Yatsunenko T, Manary MJ, et al Gut microbiomes of Malawian twin pairs discordant for kwashiorkor. Science 2013; 339:548–54. 23363771 10.1126/science.1229000 PMC3667500

[2] Xiao Y, He X, Zhang H, et al Washed microbiota transplantation effectively improves nutritional status in gastrointestinal disease-related malnourished children. Nutrition 2025; 132:112679. 39862808 10.1016/j.nut.2024.112679

[3] Subramanian S, Huq S, Yatsunenko T, et al Persistent gut microbiota immaturity in malnourished Bangladeshi children. Nature 2014; 510:417–21. 24896187 10.1038/nature13421 PMC4189846

[4] Turnbaugh PJ, Hamady M, Yatsunenko T, et al A core gut microbiome in obese and lean twins. Nature 2009; 457:480–4. 19043404 10.1038/nature07540 PMC2677729

[5] Murray CJL, Aravkin AY, Zheng P, et al Global burden of 87 risk factors in 204 countries and territories, 1990–2019: a systematic analysis for the Global Burden of Disease study 2019. Lancet 2020; 396:1223–49. 33069327 10.1016/S0140-6736(20)30752-2 PMC7566194

[6] Woodworth MH, Hayden MK, Young VB, Kwon JH. The role of fecal microbiota transplantation in reducing intestinal colonization with antibiotic-resistant organisms: the current landscape and future directions. Open Forum Infect Dis 2019; 6:ofz288. 31363779 10.1093/ofid/ofz288 PMC6667716

[7] Iddrisu I, Monteagudo-Mera A, Poveda C, et al Malnutrition and gut microbiota in children. Nutrients 2021; 13:2727. 34444887 10.3390/nu13082727 PMC8401185

[8] Gupta A, Khanna S. Fecal microbiota transplantation. JAMA 2017; 318:102. 28672320 10.1001/jama.2017.6466

[9] Rodrigues T, Rodrigues Fialho S, Araújo JR, Rocha R, Moreira-Rosário A. Procedures in fecal microbiota transplantation for treating irritable bowel syndrome: systematic review and meta-analysis. J Clin Med 2023; 12:1725. 36902512 10.3390/jcm12051725 PMC10003588

[10] Tian H, Wang X, Fang Z, et al Fecal microbiota transplantation in clinical practice: present controversies and future prospects. hLife 2024; 2:269–83.

[11] Terefe Y, Deblais L, Ghanem M, et al Co-occurrence of *Campylobacter* species in children from eastern Ethiopia, and their association with environmental enteric dysfunction, diarrhea, and host microbiome. Front Public Health 2020; 8:99. 32351922 10.3389/fpubh.2020.00099 PMC7174729

[12] Lane AA, McGuire MK, McGuire MA, et al Household composition and the infant fecal microbiome: the INSPIRE study. Am J Phys Anthropol 2019; 169:526–39. 31012086 10.1002/ajpa.23843

[13] Tesfaw G, Siraj DS, Abdissa A, et al Gut microbiota patterns associated with duration of diarrhea in children under five years of age in Ethiopia. Nat Commun 2024; 15:7532. 39223134 10.1038/s41467-024-51464-w PMC11369280

[14] Tenna A, Stenehjem EA, Margoles L, Kacha E, Blumberg HM, Kempker RR. Infection control knowledge, attitudes, and practices among healthcare workers in Addis Ababa, Ethiopia. Infect Control Hosp Epidemiol 2013; 34:1289–96. 24225614 10.1086/673979 PMC3995333

[15] Merga KH, Getachew EM, Fujita AW, et al A high prevalence of antibiotic use at two large teaching hospitals in Addis Ababa, Ethiopia: a point prevalence survey. Antimicrob Steward Healthc Epidemiol 2024; 4:e180. 39450096 10.1017/ash.2024.432 PMC11500273

[16] Hennink MM, Kaiser BN, Weber MB. What influences saturation? Estimating sample sizes in focus group research. Qual Health Res 2019; 29:1483–96. 30628545 10.1177/1049732318821692 PMC6635912

[17] Hennink MM . Assessing focus group research. In: Focus group discussions: Understanding qualitative research. Oxford: Oxford University Press, 2014:180–4.

[18] Zipursky JS, Sidorsky TI, Freedman CA, Sidorsky MN, Kirkland KB. Patient attitudes toward the use of fecal microbiota transplantation in the treatment of recurrent *Clostridium difficile* infection. Clin Infect Dis 2012; 55:1652–8. 22990849 10.1093/cid/cis809

[19] Zhang Y, Xue X, Su S, et al Patients and physicians' attitudes change on fecal microbiota transplantation for inflammatory bowel disease over the past 3 years. Ann Transl Med 2021; 9:1619. 34926663 10.21037/atm-21-3683 PMC8640917

[20] Park L, Mone A, Price JC, et al Perceptions of fecal microbiota transplantation for *Clostridium difficile* infection: factors that predict acceptance. Ann Gastroenterol 2017; 30:83–8. 28042242 10.20524/aog.2016.0098 PMC5198252

[21] Zeitz J, Bissig M, Barthel C, et al Patients' views on fecal microbiota transplantation: an acceptable therapeutic option in inflammatory bowel disease? Eur J Gastroenterol Hepatol 2017; 29:322–30. 27879485 10.1097/MEG.0000000000000783

[22] Zhong M, Sun Y, Wang HG, et al Awareness and attitude of fecal microbiota transplantation through transendoscopic enteral tubing among inflammatory bowel disease patients. World J Clin Cases 2020; 8:3786–96. 32953854 10.12998/wjcc.v8.i17.3786 PMC7479546

[23] Kahn SA, Vachon A, Rodriquez D, et al Patient perceptions of fecal microbiota transplantation for ulcerative colitis. Inflamm Bowel Dis 2013; 19:1506–13. 23624888 10.1097/MIB.0b013e318281f520 PMC3780382

[24] Park J, Hong SN, Lee HS, et al Perception of fecal microbiota transplantation in patients with ulcerative colitis in Korea: a KASID multicenter study. Korean J Intern Med 2024; 39:783–92. 39252488 10.3904/kjim.2024.053 PMC11384244

[25] Zellmer C, De Wolfe TJ, Van Hoof S, Blakney R, Safdar N. Patient perspectives on fecal microbiota transplantation for *Clostridium difficile* infection. Infect Dis Ther 2016; 5:155–64. 27048199 10.1007/s40121-016-0106-1 PMC4929085

[26] Goodman C, O’Rourke N, Amundson C, Drekonja D. Patient knowledge and attitudes about fecal microbiota therapy for *Clostridium difficile* infection. Fed Pract 2017; 34:15–9. 30766228 PMC6372037

[27] Drees M, Mcgraw P, Fogwe B, Bacon AE, Duffalo C. Attitudes toward fecal microbiota transplantation among patients with *Clostridium difficile*. Open Forum Infect Dis 2015; 2:968.

[28] Marshall DA, MacDonald KV, Kao D, et al Patient preferences for active ulcerative colitis treatments and fecal microbiota transplantation. Ther Adv Chronic Dis 2024; 15:20406223241239168. 38544906 10.1177/20406223241239168 PMC10966996

[29] Xu L, Zhang T, Cui B, et al Clinical efficacy maintains patients' positive attitudes toward fecal microbiota transplantation. Medicine (Baltimore) 2016; 95:e4055. 27472679 10.1097/MD.0000000000004055 PMC5265816

[30] Dunleavy KA, Stanley S, Hirten R, Sands BE, Grinspan AM. S0873 patient and gastroenterologist attitudes of fecal microbiota transplantation as a treatment for inflammatory bowel disease. Am Coll Gastroenterol 2020; 115:S449.

[31] Zipursky JS, Sidorsky TI, Freedman CA, Sidorsky MN, Kirkland KB. Physician attitudes toward the use of fecal microbiota transplantation for the treatment of recurrent *Clostridium difficile* infection. Can J Gastroenterol Hepatol 2014; 28:319–24. 24719899 10.1155/2014/403828 PMC4072236

[32] Al-Bakri AG, Akour AA, Al-Delaimy WK. Knowledge, attitudes, ethical and social perspectives towards fecal microbiota transplantation (FMT) among Jordanian healthcare providers. BMC Med Ethics 2021; 22:1–10. 33639935 10.1186/s12910-021-00587-6 PMC7912465

[33] Wu X, Dai M, Buch H, et al The recognition and attitudes of postgraduate medical students toward fecal microbiota transplantation: a questionnaire study. Therap Adv Gastroenterol 2019; 12:1756284819869144. 10.1177/1756284819869144 PMC6724572 31516555

[34] Moossavi S, Salimzadeh H, Katoonizadeh A, et al Physicians' knowledge and attitude towards fecal microbiota transplant in Iran. Middle East J Dig Dis 2015; 7:155–60. 26396717 PMC4560629

[35] Madar PC, Petre O, Baban A, Dumitrascu DL. Medical students' perception on fecal microbiota transplantation. BMC Med Educ 2019; 19:1–7. 31601212 10.1186/s12909-019-1804-7 PMC6788000

[36] Ma Y, Yang J, Cui B, Xu H, Xiao C, Zhang F. How Chinese clinicians face ethical and social challenges in fecal microbiota transplantation: a questionnaire study. BMC Med Ethics 2017; 18:39. 28569156 10.1186/s12910-017-0200-2 PMC5452366

[37] Ren RR, Sun G, Yang YS, et al Chinese physicians' perceptions of fecal microbiota transplantation. World J Gastroenterol 2016; 22:4757–65. 27217707 10.3748/wjg.v22.i19.4757 PMC4870082

[38] Gweon TG, Lee YJ, Yim SK, et al Recognition and attitudes of Korean physicians toward fecal microbiota transplantation: a survey study. Korean J Intern Med 2023; 38:48–55. 36353787 10.3904/kjim.2022.206 PMC9816678

[39] Gill M, Blacketer C, Chitti F, et al Physician and patient perceptions of fecal microbiota transplant for recurrent or refractory *Clostridioides difficile* in the first 6 years of a central stool bank. JGH Open 2020; 4:950–7. 33102769 10.1002/jgh3.12396 PMC7578309

[40] Mcilroy JR, Nalagatla N, Hansen R, Hart A, Hold GL. Faecal microbiota transplantation as a treatment for inflammatory bowel disease: a national survey of adult and paediatric gastroenterologists in the UK. Frontline Gastroenterol 2018; 9:250–5. 30245786 10.1136/flgastro-2017-100936 PMC6145431

[41] Chauhan U, Popov J, Farbod Y, et al Fecal microbiota transplantation for the treatment of ulcerative colitis: a qualitative assessment of patient perceptions and experiences. J Can Assoc Gastroenterol 2021; 4:e120–9. 34877470 10.1093/jcag/gwab007 PMC8643654

[42] Liu H, Wei Y, Xu Z, et al Exploring factors affecting acceptance of fecal microbiota transplantation for patients with recurrent urinary tract infections: a descriptive qualitative study. Patient Prefer Adherence 2024; 18:1257–69. 38911589 10.2147/PPA.S452328 PMC11192636

[43] Kragsnaes MS, Sødergren ST, Kjeldsen J, et al Experiences and perceptions of patients with psoriatic arthritis participating in a trial of faecal microbiota transplantation: a nested qualitative study. BMJ Open 2021; 11:e039471 10.1136/bmjopen-2020-039471 PMC7942243 34006020

[44] Liu Y, Alnababtah K, Cook S, Yu Y. Healthcare providers' perception of faecal microbiota transplantation with *Clostridium difficile* infection and inflammatory bowel disease: a quantitative systematic review. Therap Adv Gastroenterol 2021; 14:17562848211042679. 10.1177/17562848211042679 PMC8460966 34567271

[45] Bilsen MP, Lambregts MMC, van Prehn J, Kuijper EJ. Faecal microbiota replacement to eradicate antimicrobial resistant bacteria in the intestinal tract—a systematic review. Curr Opin Gastroenterol 2022; 38:15 25. 34636363 10.1097/MOG.0000000000000792 PMC8654246

[46] Saha S, Tariq R, Tosh PK, Pardi DS, Khanna S. Faecal microbiota transplantation for eradicating carriage of multidrug-resistant organisms: a systematic review. Clin Microbiol Infect 2019; 25:958–63. 30986562 10.1016/j.cmi.2019.04.006

[47] Dharmaratne P, Rahman N, Leung A, Ip M. Is there a role of faecal microbiota transplantation in reducing antibiotic resistance burden in gut? A systematic review and meta-analysis. Ann Med 2021; 53:662–81. 34170204 10.1080/07853890.2021.1927170 PMC8238059

[48] US Food and Drug Administration . FDA approves first orally administered fecal microbiota product for the prevention of recurrence of *Clostridioides difficile* infection. **2023**. Accessed 30 June 2025. Available at: https://www.fda.gov/news-events/press-announcements/fda-approves-first-orally-administered-fecal-microbiota-product-prevention-recurrence-clostridioides

