# Supplementary material for: Knowledge and Acceptability of Fecal Microbiota Transplantation Among Patients, Caregivers, and Health Care Providers in Ethiopia
Source: Open Forum Infect Dis. 2025 Nov 4;12(11):ofaf676. doi: 10.1093/ofid/ofaf676 (PMC12631766; doi:10.1093/ofid/ofaf676)
Supplement: ofaf676_Supplementary_Data [file ofaf676_supplementary_data.docx]

**Supplementary Material**

Supplementary Table 1: Demographic Information of Focus Group Participants (n=59)

| **Characteristic** | **Healthcare providers** | | **Patients/caregivers** | |
| --- | --- | --- | --- | --- |
| **Hospital** | **ALERT**  **(n=8)** | **Black Lion (n=16)** | **ALERT**  **(n=12)** | **Black Lion (n=23)** |
| **Participant sex** | | | | |
| Female | 5 | 5 | 6 | 8 |
| Male | 3 | 11 | 6 | 15 |
| **Participant role at hospital** | | | | |
| Attending physician | 5 | 3 | Not applicable | |
| Resident physician | 0 | 8 |  |  |
| Other healthcare provider (e.g., nurse, pharmacist) | 3 | 5 |  |  |

Supplementary Material 2: Patient/Caregiver Focus Group Discussion Guide

Thank you for coming today. My name is __________ and I am a researcher from (Emory University / AHRI). We are conducting a study in Ethiopia on new non-antibiotic treatments to improve common health issues. We feel that it is very important to speak to patients directly about their views on these new treatments, so we are holding these discussion groups with people like yourselves in this area. In our discussion, we will be talking about different types of new treatments, how acceptable they are to you, and what would make you more or less likely to take them.

Please don’t feel shy in the discussion as we would like to hear all your different views. Your opinions are very valuable to us, and we are here to learn from you. There are no right or wrong answers, we are simply interested in your views, so please feel comfortable to share what you think about the topics we discuss.

During the discussion, my assistant __________ will be taking notes on points we discuss, but they cannot write down everything we say, so we would like to ask you if we can record this discussion. The reason for the recording is so that we don’t miss anything you say and so the rest of the research team can also hear your views exactly. Our discussion will remain completely confidential, only the research team will listen to the recording, it will be securely stored and not accessible to anyone outside the research team. The information you give us will only be used for this research project to make the best use of these new types of treatments. **Do we have everyone’s permission to record our discussion**? (Check that all consent to recording and answer any questions). Also, we request that everything you hear today is kept confidential and not shared with anyone outside this group.

Let me tell you a little about how we would like to run this group discussion. Your participation in this group is voluntary, so if you prefer not to be part of this discussion, you are completely free to leave at any time. However, we value your opinion and hope that you will stay and share your views. We will not be going around the group for every question, so just join in when you have something to say or if you want to respond to someone else’s point, but it is also important that only one person talks at a time so that we capture everything on the recording. We want to hear as many different views as possible, so feel free to disagree with others and share your own views, but please also respect the views of everyone else here. Our discussion will last about one hour. (Please help yourselves to the refreshments). **Are there any questions before we start?**

**Let’s begin by introducing ourselves**. ***Please go around the circle and tell us where you travelled from to get here today.***

**Introduction Questions (10 minutes)**

***I would like to begin by asking about some health problems.***

1. How common is malnutrition in your community?
2. How common are infections in your community?

**Treatment Decision-making (10 minutes)**

***Let’s now discuss decision making about treatment of infections.***

1. What are the typical treatments available for infection?
2. Who decides what treatment to take for an infection or when to see a doctor about this? (probe: How is that decision made? What are some of the influences on these decisions?)
3. If a doctor prescribes a medicine/treatment for infection, what are some of the reasons for not taking it?
4. What are the typical treatments available for malnutrition?
5. In a household, who decides what treatment to take for child malnutrition or when to see a doctor about this? (probe: How is that decision made? What are some of the influences on these decisions?)
6. If a doctor prescribes a medicine/treatment for malnutrition, what are some of the reasons for not taking it?

**Microbiome Therapeutics (MT)**

***I’d like us to now discuss a new type of treatment for infection and malnutrition called microbiome therapeutics (MT for short). These MT medicines are usually made from bacteria collected from healthy people. Some people think of them like yogurt. Some MT medicines work better than antibiotics for some types of infections and may be effective for severe malnutrition.***

1. What have you heard about MT treatment, if anything? (probe: where did you hear this?)
2. How acceptable would MT treatment be in your community if people learned it was made from stool?
3. How acceptable would MT treatment be in your community if people learned it was made from bacteria ? (probe: which is more acceptable? Why ?)
4. How acceptable would MT treatment be in your community if it was better than antibiotics or other treatments?
5. What questions would you have for a doctor about MT medicines if they told you it was the best treatment for you? (probe: How likely would you be to take an MT medicine made from stool if a doctor answered all of your questions and said it was safe?)
6. What would make you more comfortable to accept MT treatment made from stool?
7. What are some concerns about using MT treatment made from stool?

**MT Formulations**

***Let’s talk about some ways that MT medicines can be given and what might make them easier to take. MT medicines can be administered in different ways, but most commonly as capsules (refer to picture or sample capsule).***

1. What are your concerns about taking MT medicine in capsule form (probe: Color? Size? Smell/Taste? Number of capsules? At home or at a doctor’s office?)
2. What would you want to know about how the stool bacteria in MT are collected? (probe: characteristics of stool donor)

***We are coming to the end of our discussion, I have just a few last questions****.*

1. Overall, what might be the ONE main reason that someone would not want to take an MT?
2. What might be the ONE main reason that someone would want to take an MT?

***Thank you for your time and contribution to this research.***

Supplementary Material 3: HCP Focus Group Discussion Guide

Thank you for coming today. My name is __________ and I am a researcher from (Emory University / AHRI). We are conducting a study in Ethiopia on new non-antibiotic treatments to improve health. These non-antibiotic treatments are called microbiome therapeutics (MT) and involve administering mixtures of live-bacteria. We feel that it is very important to speak to doctors and healthcare providers directly about their experiences, so we are holding these discussion groups with people like yourselves to hear your views. In our discussion, we will be talking about different types of MT, how acceptable they are to you and your patients, and what would make you more or less likely to prescribe them.

Please don’t feel shy in the discussion as we would like to hear all your different views. Your opinions are very valuable to us, and we are here to learn from you. There are no right or wrong answers, we are simply interested in your views, so please feel comfortable to share what you think about the topics we discuss.

During the discussion, my assistant __________ will be taking notes on points we discuss, but they cannot write down everything we say, so we would like to ask you if we can record this discussion. The reason for the recording is so that we don’t miss anything you say and so the rest of the research team can also hear your views exactly. Our discussion will remain completely confidential, only the research team will listen to the recording, it will be securely stored and not accessible to anyone outside the research team. The information you give us will only be used for this research project to better understand perceptions of MT. **Do we have everyone’s permission to record our discussion**? (Check that all consent to recording and answer any questions). Also, we request that everything you hear today is kept confidential and not shared with anyone outside this group.

Let me tell you a little about how we would like to run this group discussion. Your participation in this group is voluntary, so if you prefer not to be part of this discussion, you are completely free to leave at any time. However, we value your opinion and hope that you will stay and share your views. We will not be going around the group for every question, so just join in when you have something to say or if you want to respond to someone else’s point, but it is also important that only one person talks at a time so that we capture everything on the recording. We want to hear as many different views as possible, so feel free to disagree with others and share your own views, but please also respect the views of everyone else here. Our discussion will last about one hour. (Please help yourselves to the refreshments). **Are there any questions before we start?**

**Let’s begin by introducing ourselves**. ***Please go around the circle and tell us your profession?***

**Introduction Questions (10 minutes)**

***I would like to begin by talking about some different types of health problems.***

1. How common is child malnutrition amongst your patients?
2. How common are infections caused by antibiotic resistant bacteria amongst your patients?

**Microbiome Therapeutics (MT)**

***I’d like us to now discuss a new type of medicine called microbiome therapeutics (MT for short). These MT are usually made from bacteria and/or stool collected from healthy people. Some people think of them like yogurt. Some MT medicines work better than antibiotics for some types of infections and may be effective for severe malnutrition.***

Providers Perceptions

1. What have you heard about MT treatment, if anything? (probe: source of knowledge)
2. How acceptable is prescribing MT treatment among providers like yourself knowing it was made from stool and/or bacteria (probe: is one more acceptable than the other stool vs. cultured bacteria and why?)
3. How likely would you be to prescribe an MT if it was better than antibiotics or other available treatments to prevent infections with antibiotic resistant bacteria?
4. How likely would you be to prescribe a stool-derived MT if it was as safe as antibiotics?
5. What would make you more comfortable to prescribe MT treatment made from stool? (probe: data, incorporation in guidelines, peer prescribing, patient preference, accessibility)
6. What are some concerns about prescribing MT treatment made from stool? (probe: safety, efficacy, patient preference, accessibility, cultural norms)

**MT Perceptions and Formulations**

***Let’s talk about some ways that MT medicines can be given, what might make them easier to take and how patients may perceive them. MT medicines can be administered in different ways, but most commonly as capsules (refer to picture or sample capsule).***

1. How likely do you think your patients would be to take an MT knowing it is made from stool and/or live bacteria? (probe: how important is it to share the composition of the MT with patients?, is one more acceptable than the other [stool vs. cultured bacteria] and why ?)
2. What are your concerns about patients taking MT medicine in capsule form? (probe: Color? Size? Smell/Taste? Number of capsules? repeat dosing? At home or at a doctor’s office?
3. What are your concerns about patients taking MT medicine in non-capsule form (eg. NG-delivered formulations or enema? (probe: feasibility, infrastructure, time, staffing, access, patient preference?)
4. What would you want to know about the donor for MT medicines made from stool? probe: (age, sex, health status, race, local vs. non-local?)

***We are coming to the end of our discussion, I have one last question****.*

1. Overall, what is the most important reason providers like yourself would want to prescribe an MT?

***Thank you for your time and contribution to this research.***
